# Clinical case report: considerable improvement of severe and difficult-to-treat obsessive-compulsive disorder with comorbid depression under treatment with esketamine and concomitant psychotherapy

**DOI:** 10.3389/fpsyt.2023.1291077

**Published:** 2023-11-28

**Authors:** Alexander Kaltenboeck, Elisa Foerster, Sabrina Strafner, Ulrike Demal, Nilufar Mossaheb, Fabian Friedrich

**Affiliations:** ^1^Clinical Division of Social Psychiatry, Department of Psychiatry and Psychotherapy, Medical University of Vienna, Vienna, Austria; ^2^Division of Clinical Psychology and Psychotherapy, Medical Head Office (Ärztliche Direktion), University Hospital Vienna, Vienna, Austria

**Keywords:** obsessive-compulsive disorder, depression, ketamine, ketamine-assisted psychotherapy, case report

## Abstract

A 28-year-old man was admitted to our psychiatric ward with severe obsessive-compulsive disorder (OCD) and comorbid depression. At intake, obsessive-compulsive symptoms were present most time of the day and were related to an intense fear of causing interpersonal misunderstandings. Various treatment attempts, including cognitive-behavioural therapy (CBT) with exposure and response prevention (ERP), selective serotonin reuptake inhibitors, clomipramine, and add-on antipsychotics were either ineffective and/or were not tolerated, and the patient’s condition worsened progressively. Following an in-depth multidisciplinary team discussion and a shared decision-making process, an off-label treatment trial with esketamine and concomitant psychotherapy was started. The patient received 10 esketamine + psychotherapy sessions over a period of about 2 months, followed by two maintenance sessions in about 3-week intervals. After this, he was discharged into regular outpatient care where he continued to receive maintenance esketamine treatment every 4–6 weeks and, independent of this, individual CBT. Following the establishment of esketamine with concurrent psychotherapy, the patient exhibited a remarkable clinical improvement. Obsessive-compulsive symptoms showed a clear and sustained response (Y-BOCS before treatment >35, Y-BOCS at week 8 = 23, Y-BOCS at week 26 = 14). Paralleling this, depressive symptoms also decreased (MADRS before treatment = 47, MADRS at week 9 = 12, MADRS at week 26 = 3). At a naturalistic follow-up at week 66, obsessive-compulsive symptoms were still mild (Y-BOCS = 13), and depression was still in remission (MADRS < 6). This clinical case suggests that (es)ketamine plus concomitant psychotherapy may hold promise as a therapeutic strategy for difficult-to-treat OCD and depression and its full clinical potential should be evaluated in more comprehensive future studies.

## Introduction

Obsessive-compulsive disorder (OCD) is a common, severe, and chronic mental health condition that can lead to a significant impairment in quality of life (1, 2). Its symptoms — intrusive and distressing obsessions and/or compulsive behaviours and rituals — can interfere considerably with day-to-day functioning (2). Standard pharmacological treatment approaches to the condition include selective serotonin reuptake inhibitors (SSRIs) and/or clomipramine, as well as augmentation with atypical antipsychotics (especially aripiprazole and risperidone) (2, 3). The most established evidence-based psychotherapeutic treatment is cognitive behavioural therapy (CBT) with exposure and response prevention (ERP), whereby the patient is deliberately exposed to OCD symptom-provoking situations, whilst refraining from engaging in compulsive behaviours (2, 3). Despite the efficacy of these first-line treatment options in many patients, there is still a considerable proportion (estimated ~30%) of people whose OCD symptoms do not improve significantly over time despite adequate treatment (4–6). Such difficult-to-treat cases of OCD can be exceptionally challenging in clinical practice and there is an urgent need for novel interventions that can be offered to affected patients.

A novel therapeutic avenue to explore might be treatment with ketamine, which has attracted considerable attention for its therapeutic potential in major depression (7). Both, ketamine and its enantiomer esketamine, have been documented to lead to rapid improvement of depressive symptoms, even in patients who have previously not responded to standard antidepressant treatments and regulatory authorities in various countries have now approved an intranasal esketamine formulation for treatment-resistant depression (7). This therapeutic success has spurred further research into ketamine’s therapeutic potential for other common psychiatric conditions such as OCD (8–11).

One interesting therapeutic approach that has recently gained momentum is the combination of ketamine treatment with concurrent psychotherapy in order to maximize and prolong the (often only short-lived) clinical improvements brought about by administration of ketamine alone (12, 13). Preliminary research indeed supports the idea that psychotherapy before, during, or following ketamine sessions can induce and extend clinical improvements in depression, anxiety, and addiction and further therapeutic rapport and treatment engagement (12, 13).

Here, we report on a 28-year-old patient with severe, treatment-resistant OCD and comorbid depression who received off-label treatment with esketamine combined with psychotherapy and showed a remarkable clinical response. The purpose of the report is to describe the clinical use of repeated esketamine + psychotherapy sessions and their effects, to highlight their potential, and to motivate further research into this therapeutic avenue.

## Clinical presentation

Mr. B., a 28-year-old man with a long-standing history of OCD and recurrent depressive disorder, was admitted as an inpatient to our psychiatric clinic because of recent aggravation of obsessive-compulsive and depressive symptoms.

OCD symptoms had first emerged during Mr. B.’s early adolescence and had thematically evolved over the course of his life, including excessive worrying about forgetting or missing something important (e.g., forgetting to switch off electrical appliances or to lock car doors), preoccupation with and intrusive repeating of specific numbers (e.g., the current time), recurrent mental and behavioural checking of things said and done, and repetitive hand washing. Mr. B. had undergone a range of different psychopharmacological and psychological treatments throughout his life. He had received psychiatric and psychotherapeutic outpatient treatment for several years, had been admitted as a psychiatric inpatient multiple times, and had twice completed a specialised 8-week inpatient programme for OCD that encompassed psychopharmacological management and intensive group and individual CBT with ERP. Obsessive-compulsive symptoms had waxed and waned with different treatments but had never reached remission and in the last months had progressively worsened. Paralleling the severity of obsessive-compulsive symptoms, Mr. B. had also experienced multiple episodes of major depression throughout his life and had attempted suicide at least three times, always related to obsessive-compulsive symptom burden. The last suicide attempt had taken place less than a month before admission to our ward. Physical examinations, laboratory testing, and cranial magnetic resonance imaging had been performed multiple times since disorder onset but had never shown any significant abnormality that could be causally linked to obsessive-compulsive or depressive symptoms.

At intake, Mr. B.’s OCD symptoms centred around an intense fear of being responsible for interpersonal misunderstandings, which led to continuous mental reviewing of conversations, excessive interpersonal reassurance-seeking, and the performance of various time-demanding ritualistic behaviours, such as repeating verbal statements multiple times whilst talking to another person. In addition, Mr. B. also showed compulsive hand washing and spent a considerable time ruminating about how to best wash certain body parts. Obsessive-compulsive symptoms affected not only the patient himself, but also significantly interfered with how he could interact with his loved ones, fellow patients, and his treatment team. Mr. B. indicated several times that he avoided social contact because of feeling ashamed of his bizarre-appearing obsessive-compulsive symptoms. Along with the worsening of OCD, Mr. B. had also developed a range of depressive symptoms which, at the time of presentation, included low mood, decreased interest and pleasure, loss of energy, hypersomnia, diminished ability to concentrate, indecisiveness, feelings of worthlessness, loss of appetite, and occasional albeit recurrent passive suicidal ideation.

We conducted a full psychiatric workup, which included a detailed history obtained from the patient (complemented by collateral information from family members as well consultation of discharge papers from previous psychiatric/psychotherapeutic treatments), standard physical and neurological examinations, electrocardiography, standard laboratory blood tests including measurement of drug levels and pharmacogenetic testing, and cranial magnetic resonance imaging. We found no conclusive evidence for important differential diagnoses such as bipolar disorder, a psychotic disorder, a substance-related disorder, a neurocognitive disorder, a mental health problem due to a substance or a medical condition, or a neurodegenerative process. We also found no clear reason for the lack of an effect of standard psychopharmacological treatment.

At the time of admission, Mr. B. was on a psychopharmacological regimen for OCD and depression comprising sertraline 200 mg/d, risperidone 3 mg/d, quetiapine 150 mg/d, and quetiapine extended release 200 mg/d. Because of increasing agitation and anxiety, suicidal ideation and sleeping difficulties, his previous psychiatric treatment team had additionally started him on lorazepam 2 mg/d and prothipendyl 120 mg/d. Mr. B. had been on treatment with sertraline, risperidone, and quetiapine for more than 8 weeks without any significant effect on OCD or depression. According to patient history, two past treatments with SSRIs (fluoxetine and escitalopram) had not shown a lasting effect either and a trial with add-on aripiprazole had not been tolerated.

We initially opted for a trial with add-on clomipramine to target both obsessive-compulsive and depressive symptoms. Risperidone was stopped (due to low tolerability) and quetiapine and quetiapine extended release were increased in the course of treatment to a maximum of 200 mg/d and 300 mg/d, respectively. Clomipramine was titrated up to 150 mg/d and sertraline was increased to 250 mg/d. Under this medication, however, Mr. B. developed severe serotonergic side effects, including nervousness, tremor, hyperreflexia, myoclonus, and muscular rigidity. These only ceased after clomipramine was stopped and sertraline was reduced back to 200 mg/d. Because of worsening depressive symptoms, bupropion extended release 150 mg/d was started, which, according to patient history, had previously been helpful for depressive symptoms. Although his clinical history was not positive for epilepsy, Mr. B. developed a seizure a few days after treatment with bupropion had been started. Following a full neurological workup, epilepsy was diagnosed by the consulting neurology team, bupropion extended release was stopped, and anti-seizure medication (first levetiracetam, then switched to sodium valproate) was started.

Following the seizure, the patient’s psychological condition further deteriorated. Because of continuously aggravating obsessive-compulsive symptoms (especially repeating of sentences in conversations and nearly constant reassurance-seeking), it became almost impossible for him to engage in fluent communication, which progressively interfered with his ability to take part in individual psychotherapy. The patient spent most of the day either engaged in obsessions and compulsive behaviour or sleeping to avoid having to confront these symptoms. Mr. B. grew desperate and suicidal ideation became more pressing and concrete.

Facing the complexity of the situation (lack of clinical effect and/or intolerance of multiple previous pharmacological trials, significant impairment to partake in psychotherapy, OCD and depression progressively worsening, risk of suicide escalating), we initiated an in-depth, multidisciplinary case discussion and evaluated further therapeutic options together with the patient and his family. As a result of this, following a shared-decision process, an off-label treatment trial with esketamine and concomitant psychotherapy was started, considering that our previous diagnostic workup (see above for details) had not shown any contraindications.

Mr. B. received eight intravenous esketamine treatments at a dose of ~0.35 mg/kg over a period of 8 weeks (including 3 weeks treatment break for discharge over Christmas holidays). Esketamine administration was then switched to intranasal route to prepare for eventual maintenance treatment outside our clinic. Mr. B. received two more intranasal esketamine treatments at a dose of ~0.8 mg/kg, once a week, and was then discharged in a stable and improved mental state. He received two further maintenance treatments sessions with intranasal esketamine (at a dose of ~1.2 mg/kg) in about 3-week intervals at our clinic. He was then referred to regular psychiatric outpatient treatment, where maintenance esketamine treatment every 4–6 weeks with regular re-evaluation of treatment need was established.

Esketamine treatment at our ward was complemented by concomitant psychotherapy. This included elements of psychoeducation and metacognitive training, CBT with ERP, and mindfulness-based interventions and was conducted both during acute esketamine treatment and in-between esketamine sessions. For safety reasons, during esketamine + psychotherapy sessions two clinicians (one psychiatrist and one psychologist) were present with the patient. Both received regular intervision sessions with the rest of the multidisciplinary team to discuss the progress of the treatment and any potential difficulties that might arise. Due to the severity of OCD symptoms, especially at the beginning of treatment, psychotherapy had to be heavily adjusted to allow for meaningful interaction with the patient. The first esketamine + psychotherapy sessions were more focused on getting the patient acquainted with the drug’s subjective effects and supporting him in noticing and savouring any acute positive effects that became apparent. Gradually, more complex psychotherapeutic interventions, such as cognitive restructuring and ERP could be conducted during esketamine treatment. Between esketamine + psychotherapy sessions, the patient also had regular individual psychotherapy sessions that aimed to integrate experiences made during esketamine treatment and to solidify and expand clinical improvements. All esketamine + psychotherapy sessions were well-tolerated, with side effects of acute esketamine treatment as to be expected (mainly dizziness, dissociation, and sedation).

Quantitative assessment of symptoms was done using the Clinical Global Impression rating scale (CGI), Yale-Brown Obsessive-Compulsive Scale (Y-BOCS), and Montgomery-Åsberg Depression Rating Scale (MADRS). Pre-treatment ratings indicated a high symptom burden for both, OCD and depression. Y-BOCS could not be completed with the patient prior to treatment because of extreme compulsive repeating and reassurance-seeking when talking about OCD symptoms, but clinician estimated Y-BOCS was >35 and CGI OCD = 7. Depression ratings were similarly severe with MADRS = 47 and CGI depression = 6. After the first esketamine infusion, Mr. B. reported a brief anti-obsessional and anti-depressive effect, lasting for about 1 h. After two further esketamine + psychotherapy sessions, Mr. B.’s clinical improvements began to last longer, and he was able to engage more fluently in psychotherapy sessions again. Interacting with him became easier, which both the patient himself and his family noticed as a clear improvement. The patient was then temporarily discharged at his own request for 3 weeks to spend time with his family over the Christmas holidays.

After this break, Mr. B. was readmitted and esketamine + psychotherapy treatment was continued. In addition, his psychopharmacological medication was slightly adjusted. Pregabalin was started and titrated up to 600 mg/d to tackle the patient’s tendency for generalised anxiety and worrying. In exchange, quetiapine immediate and extended release were tapered down. We initially left quetiapine extended release at 50 mg/d at the patient’s request, as he found it helpful for occasional sleeping difficulties, but in the course of continued treatment this could later be stopped as well. Following further clinical improvement, benzodiazepine treatment could also be stopped.

About 2 months after treatment with esketamine and concomitant psychotherapy had begun, significant changes compared to pre-treatment scores were measurable in both OCD symptoms (Y-BOCS = 23, CGI OCD = 4) and depressive symptoms (MADRS = 12, CGI depression = 2). After 10 esketamine + psychotherapy sessions in total, we discharged Mr. B. in a stable and improved mental state. Two further maintenance treatment sessions at intervals of about 3 weeks were scheduled at our clinic. At intake for the first maintenance session, the patient’s symptoms had worsened (Y-BOCS = 32, CGI OCD = 4, MADRS = 21, CGI depression = 3). The patient linked this to subjectively overwhelming psychosocial demands following discharge from a relatively long hospital stay. At the next intake for maintenance treatment about 3 weeks later, symptoms had improved again (Y-BOCS = 21, CGI OCD = 4, MADRS = 11, CGI depression = 2).

Following his discharge from the last maintenance session at our clinic, Mr. B. continued to receive external outpatient psychiatric and psychotherapeutic treatment, including intranasal esketamine every 4–6 weeks, individual CBT, and pharmacological treatment as had been recommended by our team (i.e., sertraline 200 mg/d, sodium valproate 1,000 mg/d, pregabalin 600 mg/d).

At a follow-up meeting about 10 weeks after discharge, depression had remitted (MADRS = 3, CGI depression = 1) and OCD had further improved (Y-BOCS = 14, CGI OCD = 3). Mr. B. indicated that he was about to start working again, attended outpatient psychotherapy on a regular basis, and adhered to psychopharmacotherapeutic treatment that still included regular maintenance esketamine sessions in an outpatient psychiatric practice. At that time, the patient had also taken part in a documentary about OCD that was broadcasted on national television where he had given a fluent face-to-face interview talking about his experience with OCD and his esketamine + psychotherapy treatment. Mr. B. summarised his subjective experience of the esketamine + psychotherapy intervention as follows:

“Then I got [esketamine] via an infusion with concomitant psychotherapy and that finally brought the therapeutic breakthrough, which actually allowed me to fully engage in psychotherapy in the first place, because the verbal OCD symptoms had hindered me a lot. Of course, my condition did not improve from one day to the next, but gradually my mood and energy became significantly better. And the OCD symptoms improved too. […] I was able to verbally get into contact with my environment again. This feeling, to finally be able to speak fluently again, was indescribable. For me, it was an enormous relief to communicate with other humans like I used to. […] Simply this experience of success, to be somewhat mentally stable again and to be able to cope with daily life, was truly breathtaking.”

**Figure 1:**
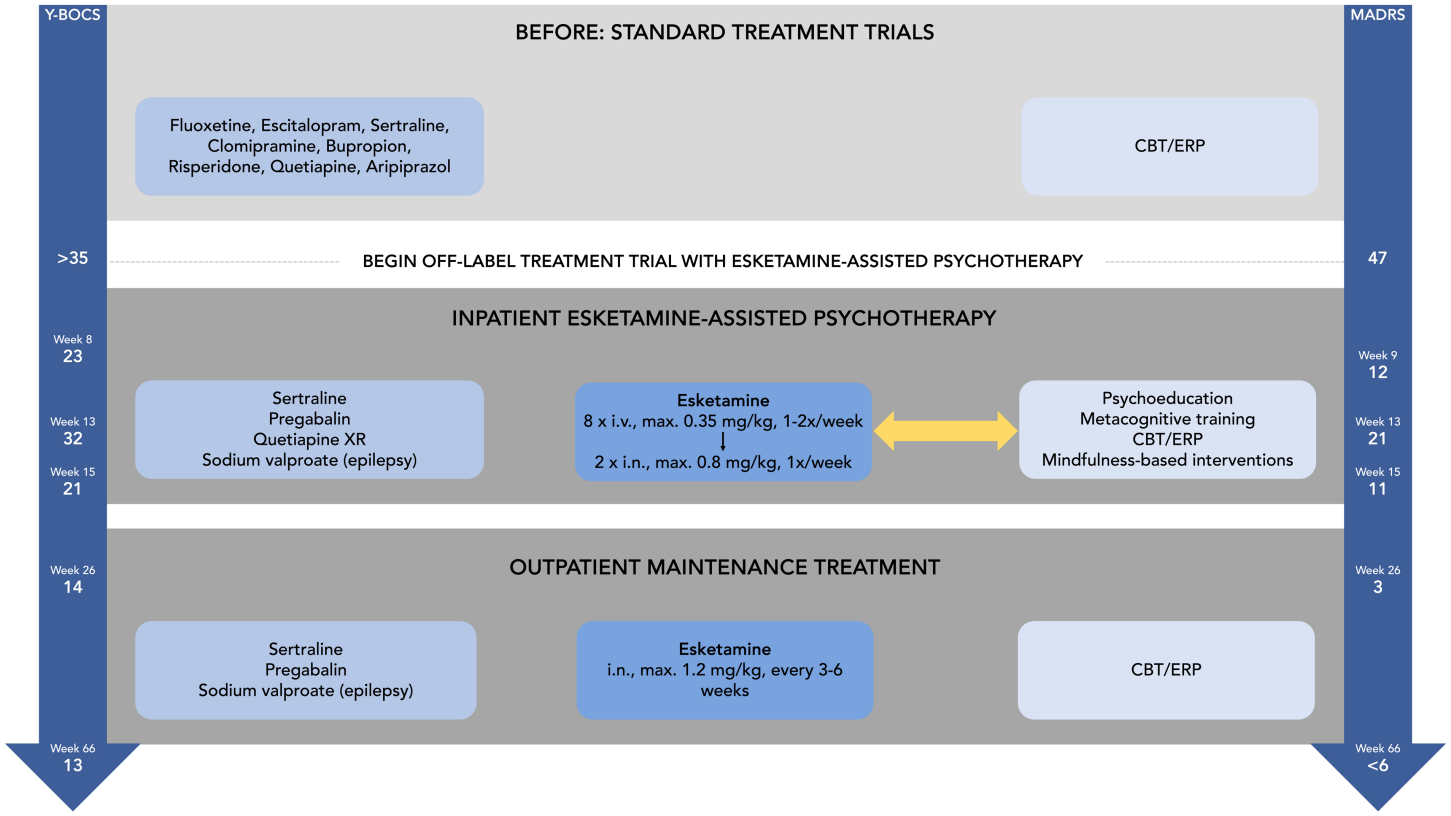
Schematic overview of Mr. B.’s treatment and clinical development.

For a schematic overview of Mr. B.’s inpatient treatment and clinical development, please refer to Figure 1.

## Discussion

Here, we report on the clinical case of a 28-year-old patient with severe, difficult-to-treat OCD and depression who, despite having undergone multiple unsuccessful first-line treatment attempts, experienced a considerable and enduring clinical response under treatment with esketamine and concomitant psychotherapy.

Our clinical observation is consistent with a growing body of literature suggesting that ketamine treatment is an efficacious intervention for patients with OCD (8, 9). Whilst previous research efforts have focused primarily on single-dose racemic ketamine treatment in this patient group (7, 8), the case of Mr. B. indicates that ketamine’s (S)-enantiomer can also have beneficial effects in OCD and that symptom improvements can be upheld with repeated treatment sessions. Considering the complexity of this case — including the chronicity and severity of obsessive-compulsive symptoms, the lack of a clinical effect and intolerance of multiple psychopharmacological trials, and the increasing inability to partake in psychotherapy due to OCD symptoms — the magnitude of the clinical response we observed is striking. This suggests that repeated esketamine treatment (with or without concurrent psychotherapy) deserves closer investigation as a potential intervention for OCD.

Another important aspect to highlight about this case, is the concurrent use of esketamine with psychotherapy. From a mechanistic viewpoint, ketamine seems well-suited for combination with psychological interventions. It modulates neurotransmitter systems (e.g., glutamate) that have been implicated in learning, belief-updating, and memory (14, 15). Furthermore, ketamine is thought to induce neuroplasticity and support cognitive flexibility, so it could open up a unique window of opportunity for psychotherapeutic interventions (15, 16). Indeed, combined treatment with ketamine and CBT with ERP as a therapeutic approach to OCD has been trialled in a small number of patients by other authors before, showing promising initial results (17, 18). To our understanding, these patients received CBT with ERP following esketamine treatment, but not whilst experiencing the acute thought-dissolving and psychodysleptic effects of the drug (14). However, these latter effects have previously been hypothesised to be particularly useful for ERP (14). The case of Mr. B. demonstrates that psychotherapy including elements of psychoeducation and metacognitive training, CBT with ERP, and mindfulness-based practices can indeed be safely and effectively used in conjunction with esketamine treatment — not only in-between esketamine sessions, but also whilst a patient receives the drug and experiences its acute effects. Indeed, in our opinion it was especially during esketamine treatment that the patient was able to engage in psychotherapeutic work that would otherwise not have been possible because of interference by extreme OCD symptoms.

Several important limitations to the interpretation of this case report should be noted. As with every description of a single case, no generalizations can be made and a direct causal connection between the treatment and the observed clinical changes cannot be proven. Neither can we rule out a placebo effect or other influences that improved the patient’s condition (e.g., therapeutic setting, concurrent psychopharmacological and psychotherapeutic treatment) — though considering the clinical time course, we find this rather unlikely. Further, OCD and depression improved simultaneously, and the relationship between these two conditions in the patient remains unclear. Thus, the improvement of obsessive-compulsive symptoms could also, at least in part, be due to ketamine-induced alleviation of depression. Finally, due to the experimental nature of the treatment, no prognosis can be made. During follow-up visits over a period of about 50 weeks after his discharge, Mr. B. remained in good overall mental and physical health and reported no relevant adverse effects of treatment.

To conclude, this case report highlights that esketamine treatment, especially in combination with psychotherapy, might have therapeutic potential for people with OCD and depression who do not respond to, or do not tolerate established first-line treatments. Given the considerable clinical improvement we observed in our patient following the establishment of esketamine + psychotherapy sessions, we suggest that further research should be dedicated to more comprehensively assess the efficacy, safety and potential mechanisms of action of this novel therapeutic approach to OCD.

## Data availability statement

The original contributions presented in the study are included in the article, further inquiries can be directed to the corresponding author.

## Ethics statement

Ethical approval was not required because this is a single, retrospective clinical case description. The study was conducted in accordance with local legislation and institutional requirements. Written informed consent was obtained from the patient for the publication of his clinical case including any potentially identifiable data included in this article.

## Author contributions

AK: Conceptualization, Data curation, Methodology, Project administration, Writing – original draft, Writing – review & editing. EF: Project administration, Writing – original draft, Writing – review & editing. SS: Conceptualization, Data curation, Methodology, Writing – review & editing. UD: Methodology, Supervision, Writing – review & editing. NM: Methodology, Resources, Supervision, Writing – review & editing. FF: Methodology, Resources, Supervision, Writing – review & editing.
